# Development of well-being after moving to telework: A longitudinal latent class analysis

**DOI:** 10.3389/fpsyg.2023.1098336

**Published:** 2023-03-03

**Authors:** Friedrich Kröner, Andreas Müller

**Affiliations:** Institute of Psychology, Work and Organizational Psychology, University of Duisburg-Essen, Essen, Germany

**Keywords:** telecommuting, COVID–19, work design, flexible work arrangements, job resources, longitudinal assessment

## Abstract

**Introduction:**

Due to the COVID-19 pandemic, teleworking suddenly became a reality for many individuals. Previous research shows that there are contradictory consequences of telework on well-being: while workers have the opportunity for self-directed work, intensified work behavior as well as longer hours being worked might occur at the same time. We expect that the effects of telework vary over time and may be able to explain these contradictions. Moreover, from the perspective of the job demands-resources model besides job resources, personal resources may be relevant. The aim of this study is to investigate how the mental well-being of workers unfolds over time after the onset of the pandemic and the role of telework in this process. Additionally we seek to identify the impact of available job resources and personal resources in this extraordinary situation.

**Methods:**

Data were collected online from 642 participants in Germany beginning in March 2020, with 8 weekly followup surveys. Mental well-being was measured using the WHO-5 well-being index. For personal resources we looked at occupational self-efficacy; job resources were flexible working hours, job autonomy, and social support. Job demands were telework and work intensification. First we used a group-based trajectory analysis approach to identify different well-being trajectories. Second we applied multinomial regression analysis to identify T1 predictors of well-being trajectory group membership and their interactions.

**Results:**

We found three groups of mental well-being trajectories: low, medium, and high. Their progress through the investigation period was rather stable: we observed only slight improvements of mental well-being for the high well-being group and a slight deterioration for the other two groups. Only the job demand work intensification and the personal resource occupational self-efficacy had a significant relationship to group assignment. Additionally we found interactions of telework with work intensification and occupational self-efficacy indicating a buffering mechanism of telework on the consequences of high work intensification; and low occupational self-efficacy.

**Discussion:**

Telework appears to be a useful resource that buffered high work intensification and compensated for low personal resources during the pandemic. Since data were from self-reports of a convenience sample we can't assume generalization of our results nor absence of common-method bias.

## 1. Introduction

Over the past decades, the improvement of information and communication technology (ICT) and internet access has led to an increase in the adoption of telework policies (Milasi et al., 2021). Telework, also known as telecommuting or remote work, refers to the use of ICT to perform work away from a central location (Qvortrup, 1998). Previous research has shown that telework can have both positive and negative effects on mental well-being. On one hand, teleworkers often report greater autonomy in terms of timing and scheduling their work. On the other hand, there may be risks of increased work intensification and longer work hours (Dimitrova, 2003; Weinert et al., 2014). Matusik and Mickel (2011) suggest that the pressure to be constantly accessible due to ICT use may contribute to this intensification of work. In addition to work overload and work-home conflicts, telework may also lead to feelings of social isolation and information deficit, which can contribute to feelings of exhaustion (Weinert et al., 2014; Wang et al., 2021). However, there is limited research on the long-term effects of telework on mental well-being and the psychological processes involved in how individuals adapt to telework. Taking a job demands-resources perspective, this study aims to investigate the effects of the pandemic-related switch to teleworking on well-being in a cross-occupational sample over several weeks, using a time series design.

The aim of the present study is to contribute to the understanding of the effects of telework on well-being during the initial months of the COVID-19 pandemic by investigating how the mental well-being of teleworkers unfolds over time after the onset of telework; using a person-centered approach, allowing us to identify different well-being trajectories (Howard and Hoffman, 2018). Group-based trajectory modeling (GBTM) is a useful tool for examining trajectory profiles and understanding how well-being changes over time (Nagin and Odgers, 2010). GBTM allows us to identify distinct latent classes or subgroups within a population that have different patterns of change and to understand the potential drivers of these trajectories. By acknowledging and examining differing levels of trajectory profiles using GBTM, we can gain a better understanding of the complex factors that influence well-being and identify more targeted and effective approaches for improving well-being outcomes.

The COVID-19 pandemic has highlighted the need for further research on the long-term effects of telework and other flexible working arrangements on well-being (Wang et al., 2021). Previous research has called for multi-wave and longitudinal studies to examine how telecommuting outcomes change over time (Bélanger et al., 2013; Shifrin and Michel, 2022). The current study aims to contribute to this body of knowledge by examining the working situation of teleworkers in the context of the COVID-19 pandemic. Given the sudden and widespread implementation of nationwide contact restrictions in Germany in March 2020, we have the rare opportunity to study direct stressor-strain relationships in which a specific event (the pandemic) leads to a stressor (telework during the pandemic) and ultimately affects well-being (strain). In general, it can be difficult to establish direct stressor-strain relationships in occupational health psychology, as psychological variables like stress are often not directly measurable and their time of measurement may be confused with the time of their occurrence (Kelloway and Francis, 2012; Semmer and Zapf, 2018). Our study aims to shed light on these complex relationships in the context of telework during the COVID-19 pandemic.

One important aspect of this study is that it includes participants with a range of telework experience, as well as those whose jobs are better or worse suited for teleworking and who may have different preferences for working from home or on-premises. This is a departure from previous studies which may have been affected by selection bias by only including individuals who preferred to work from home (for a discussion of potential selection biases, see Delanoeije and Verbruggen, 2020). This contributes to the theoretical understanding of telework by providing a more diverse sample. In Germany for example 50% of respondents to a survey worked from home, of which more than half had no previous experience with telework at all (Ono and Mori, 2021). The pandemic accelerated many work related changes: In an effort to reduce the risk of infection, strict measures were put in place in many countries. According to Eurofound (2020) over 37% of working Europeans switched to telework during the pandemic. Although previously to the pandemic the percentage of workers with access to telework has been increasing, only about 15% of dependent workers have had experience with it, a number which soared to 40% due to the measures put in place in many countries in March 2020 (Milasi et al., 2021).

In the following sections we first develop our thoughts on possible trajectories of well-being over time and will subsequently illustrate our hypotheses regarding predictors of trajectory profile membership after the onset of pandemic induced telework building upon the job demands-resources (JD-R) model by Demerouti et al. (2001).

### 1.1. Mental well-being in telework from a job demands-resources perspective

Following the JD-R model (Demerouti et al., 2001) work characteristics for the development of work related burnout can be grouped into two broad categories: (1) job demands, which are psychological, physical, social, and organizational factors of work, which require a physical and mental effort that usually lasts for a longer period of time. Work-related demands include: emotional and physical stress, shift work and conflicts. They are not necessarily negative or cause negative effects. Rather, they can become stressors with negative consequences if coping with the demand requires a high level of effort over the long term and there is no adequate recovery phase, and (2) job resources, which represent the psychological, physical, social and organizational working conditions, which are relevant for the achievement of work-related goals, alleviate work demands, and promote personal growth and development (Demerouti et al., 2001). Two mental processes are assumed by the model: a health-impairment process, where stressors lead to emotional exhaustion in the long run and thus to impaired well-being; and a motivational process through which job resources, such as autonomy or social support, foster motivation and work engagement (Demerouti et al., 2001).

Meta-analytic evidence supports the validity of the model and its suitability to assess employee well-being (Lesener et al., 2019). According to the JD-R model job resources may buffer the impact of job demands on job strain (Bakker and Demerouti, 2007). An extension to the JD-R model is the inclusion of personal resources (Xanthopoulou et al., 2007). Personal resources, according to Hobfoll et al. (2003, p. 632), are “aspects of the self that are generally linked to resiliency”. They serve a similar function as job resources; when job resources are in short supply, personal resources can counteract that shortage (Bakker and Demerouti, 2017). Personal resources, such as occupational self-efficacy have been shown to alleviate stress (Grau et al., 2001).

#### 1.1.1. Different types of trajectories of mental well-being after stressor onset

A proposed extension of the JD-R model by Bakker and de Vries (2021) integrates the perspective of self-regulation and argues that the emergence of strain and ultimately burnout has ongoing high job demands and low job resources as its cause. According to them daily job demands result in the accumulation of strain, which may lead to the use of maladaptive and less adaptive self-regulation strategies, while job resources and key personal resources may buffer the negative impact of job strain (Bakker and de Vries, 2021). The integration of the conservation of resources (COR) theory, which highlights the importance of the availability of resources in coping with stressors, would further support this argument by suggesting that (a) a threat of a loss of resources (b) depletion of resources and (c) failure to obtain resources following the spending of previous resources can lead to stress (Hobfoll, 1989). According to the COR theory individuals with access to more resources are more likely to gain additional resources in comparison to individuals with fewer resources, who are more likely to suffer further resource losses (Hobfoll, 1989).

In the field of organizational stress research, temporal relationships between stressors and mental health can be explained through models such as the *accumulation* model, which states that strain arises from the accumulation of stressors and does not decrease even if the stressors disappear, and the *adjustment* model, which posits that stress initially leads to dysfunction, but after a while, adjustment occurs and functioning improves again despite the stressor not having been removed (Zapf et al., 1996). We will utilize these two models to anticipate potential variations in trajectories of well-being and propose that the occurrence of the following trajectory profiles: *stagnant, deteriorating*, and *improving, dynamic* may be explained by these two processes.

In the *stagnant* trajectory profile, there is no significant change in well-being over time, which may be due to the fact that members of this trajectory profile do not experience any change in the work environment; or that, following the accumulation model above by Zapf et al. (1996), lack of resources does not allow well-being to deteriorate further, or conversely, a sufficient level of resources does not allow well-being to increase further.

At the same time, an increase in stressors in the beginning of the pandemic could lead to a *deterioration* in well-being. Reasons for the *deteriorating* trajectory profile may include, among others, increased social isolation, worsened work-non-work balance, work overload, higher expectation of being available due to information and communication technology (ICT) use (Mann and Holdsworth, 2003; Matusik and Mickel, 2011; Weinert et al., 2014; Wang et al., 2021).

On the other hand, it is also conceivable that there may be individuals who report an *improvement* in their well-being during the initial weeks of the pandemic: for people in the *improving* trajectory profile the situation might lead to additional resources and less stressors and consequently to lower experience of strain. Reasons may include, among others, those which stem from higher flexibility due to telework in regard to timing ones work (Kattenbach et al., 2010), lack of commuting due to telework (Hoehner et al., 2012), less monitoring by supervisors (Groen et al., 2018).

Finally the *dynamic* trajectory profiles may be comprised of individuals who experience an initial drop in well-being, which subsequently improves again (U-shaped trajectory), following the adjustment model by Zapf et al. (1996); or the inverse, where well-being improves and then deteriorates again (inverse U-shaped trajectory). Here, for example, the first phase may have had a particularly strong impact on well-being. Over time, individuals of this trajectory profile became accustomed and their well-being improved, but since an end to the pandemic was not in sight, well-being deteriorated again.

Based on the considerations regarding the different trajectory profiles, we hypothesize that

Hypothesis 1 (H1). GBTM will identify distinct trajectories of well-being among individuals during the initial time of the pandemic.

#### 1.1.2. Job demand: Telework

Commonly telework is regarded with a resource perspective (e.g., Kossek et al., 2006; Curzi et al., 2020). In an overview reviewing 63 articles Charalampous et al. (2018) deal with the well-being of teleworkers: both job satisfaction and organizational commitment show a positive association with telework. Regarding autonomy, it is shown that teleworkers have more freedom to manage their time, but at the same time experience work intensification (Charalampous et al., 2018).

In two studies, Mann and Holdsworth (2003) showed that a higher proportion of teleworkers reported feelings of loneliness, irritability, worry, and guilt compared to office workers. There are indicators that previous experience with telework is correlated with higher life satisfaction (Ono and Mori, 2021). Empirical data shows an increase in professional isolation, as well as an increase in work and stress in relationship with pandemic induced telework (Carillo et al., 2021).

We intend to investigate the job demands caused by the switch to telework for many workers during the pandemic according to the JD-R model (Bakker and Demerouti, 2007). We believe that the specific circumstances of the COVID-19 pandemic can help to better understand the general requirements and stressors teleworkers are facing and thus we decided on categorizing telework as a job demand for this study, where many workers likely experienced teleworking for the first time (Milasi et al., 2021). In addition, recent studies (e.g., Wang et al., 2021) show that telework may be associated with challenges and issues, such as isolation, information deficits, and other difficulties.

Circumstances, personal and job resources, job characteristics, and job demands differ between people. Accordingly we hypothesize that different trajectory profiles of workers' well-being, indicating how they steered through the pandemic, should become apparent. With this study we intend to identify these profiles and subsequently search for indicators of profile membership.

Since telework, especially in the initial phase of the COVID-19 pandemic, requires adjustment to the new situation and is also associated with increased job demands (e.g., Carillo et al., 2021; Wang et al., 2021), which are associated with higher risks of impaired mental well-being (Bakker and Demerouti, 2007), we assume that

Hypothesis 2 (H2). Moving to telework is negatively associated with trajectory profiles which indicate well-being.

#### 1.1.3. Job demand: Work intensification

Green (2004) distinguishes between *scope* and *intensity* of work efforts. The former refers to the time spent at work; the latter refers to the intensity of the work during that time. According to Burchell (2002, p. 72) work intensification can be defined as “the effort that employees put into their jobs during the time that they are working.” Green (2004) names technological change as one of the reasons for the increase in work intensification; as well as management behavior that is geared toward the employees' identification with the organization; implementation of incentive systems; loss of influence of trade unions and increasing job insecurity.

While work intensification was originally a term from the economic and sociological literature, it is increasingly used in a psychological context (e.g., Kelliher and Anderson, 2010; Franke, 2015; Mauno et al., 2019). Consequences of work intensification are lower job satisfaction and increased emotional exhaustion (Kubicek et al., 2015), increased fatigue and stress, as well as disturbed work-life-balance (Macky and Boxall, 2008; Boxall and Macky, 2014).

We consider work intensification as a job demand which is amplified by telework, where ICT-use is more dominant. As such, we hypothesize

Hypothesis 3 (H3). Work intensification is negatively associated with trajectory profiles which indicate well-being.

Hypothesis 4 (H4). The experience of work intensification moderates the effect of telework; in such a way that its negative effect on well-being trajectory membership is amplified.

#### 1.1.4. Personal resource: Occupational self-efficacy

Bandura's (1977) model of self-efficacy describes the extent of expectation a person has regarding their competency to perform an action and to be able to cope with difficult situations by themselves. Self-efficacy is not a construct that is equally pronounced in all areas of life, but can be pronounced in specific areas (e.g., private vs. professional life). Occupational self-efficacy refers to the extent a person is confident in being able to manage the task at hand at work (Schyns and von Collani, 2002).

Self-efficacy is an important personal resource to maintain well-being: using a meta-analytic approach medium sized negative associations between self-efficacy and burnout across countries were identified (Shoji et al., 2016). Since work accomplishment in the telework context is to a lesser degree determined by external factors, research shows that self regulation strategies, such as self-efficacy, are particularly important (Mihalca et al., 2021). Additionally there exists evidence for a positive relationship between occupational self-efficacy and employee engagement, indicating a well-being promoting function of self-efficacy at work (Pati and Kumar, 2010).

Regarding adjustment to telework, higher levels of self-efficacy are associated with beneficial behavioral strategies as well as improved adjustment to teleworking; especially for people who spend more time teleworking this relationship is stronger, which indicates the importance of this personal resource in the context of the pandemic (Raghuram et al., 2003).

Hypothesis 5a (H5a). Personal resources (occupational self-efficacy) predict trajectory profile membership. Occupational self-efficacy is positively associated with trajectory profiles which indicate well-being.

#### 1.1.5. Job resource: Social support

Social support is regarded as one of the main job resources in general: it has been identified as a resource, which mitigates perceived stressors, reduces the experience of strains, and moderates the stressor–strain relationship (Viswesvaran et al., 1999). Moreover, recent studies indicate that social support is particularly relevant in telework because remote work separated by time and space can make it difficult to access social support: perceived organizational support and perceived social support had a negative relationship with psychological strain (Bentley et al., 2016).

Wang et al. (2021) found a positive effect of social support on challenges posed by telework during the pandemic. A Finnish study identified factors related to COVID-19 anxiety of workers: perceived loneliness, technostress, neuroticism, and psychological distress contributed, among others, to increased levels of COVID-19 anxiety and thus impaired well-being (Savolainen et al., 2021). Supervisor support in the beginning of the COVID-19 pandemic had a negative effect on perceived uncertainties of university' employees, which in turn mediate the negative effect of supervisor support on their emotional exhaustion (Charoensukmongkol and Phungsoonthorn, 2020).

#### 1.1.6. Job resource: Decision-making autonomy

Job autonomy is one key determinant of employee well-being and health in major theories of work design (e.g., job characteristics model, Hackman and Oldham, 1976; job demands-control model, Karasek, 1979; and job demands-resources model, Demerouti et al., 2001) and refers to the extent in which employees have freedom to schedule tasks, make decisions and choose work methods (Morgeson and Humphrey, 2006). According to Gajendran et al. (2015) telework is associated with favorable outcomes which can be explained by job autonomy.

#### 1.1.7. Job resource: Flexitime

Flexitime (also flextime or flexible working hours) refers to the level of employees' freedom in deciding starting and ending time of work, as well as breaks (Hill et al., 2008; Barney and Elias, 2010). Teleworkers report more flexibility in structuring their workday (Dimitrova, 2003; Wilks and Billsberry, 2007). We see flexitime as a job resource similar to decision-making autonomy, since it expresses the leeway given to them in carrying out their work.

Hypothesis 5b (H5b). Job resources (social support, decision-making autonomy, flexitime) predict trajectory profile membership. Job resources are positively associated with trajectory profiles which indicate well-being.

The negative impact of job demands may be mitigated through job resources, while interacting with them in such a way, that job resources' effect is moderated by job demands; indicating the importance of job resources when job demands are high (Bakker and Demerouti, 2007).

Hypothesis 6a (H6a). Personal resources moderate the effect of the job demand telework on trajectory profile membership; in such a way that the negative effect of telework on well-being trajectory is buffered.

Hypothesis 6b (H6b). Job resources moderate the effect of the job demand telework on trajectory profile membership; in such a way that the negative effect of telework on well-being trajectory is buffered.

**Figure 1:**
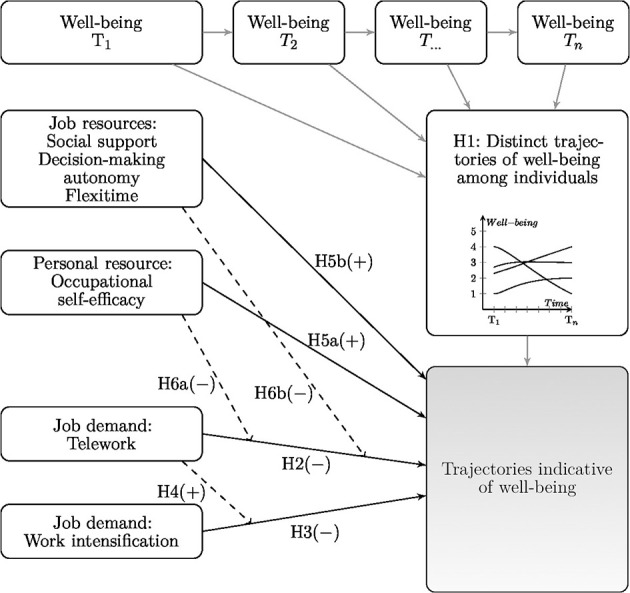
Proposed model. Dashed lines indicate moderation.

Figure 1 illustrates our research model.

## 2. Method

### 2.1. Procedure

Data analyzed in this study were collected in Germany from 2020–03–24 until 2020–05–17, shortly after the lockdown in Germany was implemented. Participants were able to register with their email address after confirming a consent form. They then received an e-mail with a link to the survey. On the survey page, demographics and job-related questions were initially collected. The next pages included items regarding working conditions, their experience during the COVID-19 situation, and well-being.

Follow-up surveys took place every week (first five surveys). At the beginning, participants were asked whether they had worked in the previous week and whether anything had changed at work. Here, the repeated survey of working conditions wasn't offered if no changes regarding their job had taken place. Participants were reminded of missed surveys at irregular intervals (up to five times). We always offered the opportunity to opt-out of participation in each invitation and reminder email.

The study received a positive assessment from the institute of psychology of the University of Duisburg-Essen's Ethics Committee.

### 2.2. Study design

In this study we used the weekly measurements of well-being to model our different groups of well-being trajectories. Job demands (such as telework and work intensification), as well as job and personal resources were measured at T1, the initial survey time point.

### 2.3. Participants

The sample consisted of 642 participants who completed the initial survey, of whom 453 (70.6%) were women. Participants had a mean age of 39.63 (*SD* = 12.81; Range: 18–79) and were mostly well-educated: Highschool or below: 21.3%; Apprenticeship: 12.3%; Bachelor: 17.1%; Master: 34.4%; Doctorate: 10.4%; Other: 4.4%. 392 (61.1%) of the participants had a full-time employment, while 183 (28.5%) worked part-time. The remaining 67 (10.4%) were in marginal and/or irregular employment or doing an apprenticeship. Job tenure of the sample was 8.12 years on average (*SD* = 9.56). One hundred sixty-five (25.7%) participants had leadership responsibilities.

While 411 (64.0%) of the participants stated, that their tasks were possible to be carried out from home, 378 (58.9%) had previous access to telework. In the initial time (T1) of the COVID-19 pandemic and its associated lockdown in Germany 403 (62.8%) of the participants were working from home. Additional descriptive statistics are available in Table 1.

**Table 1:** Sample statistics.

	**Overall**
*n*	642
Gender = Female (%)	453 (70.6)
Age [mean (SD)]	39.63 (12.81)
Children [mean (SD)]	1.77 (0.89)
Cohabitants [mean (SD)]	2.48 (1.46)
Supervisor = Yes (%)	165 (25.7)
Job tenure [mean (SD)]	8.12 (9.56)
Job status (%)	
Apprenticeship	18 (2.8)
Employed full-time	392 (61.1)
Employed part-time	183 (28.5)
In marginal or irregular employment	49 (7.6)
Employment (%)	
Apprentice/trainee/intern	15 (2.3)
Blue-collar worker	13 (2.0)
Civil servant	51 (7.9)
Freelancer/fee-based	18 (2.8)
Self-employed	34 (5.3)
White-collar worker in public sector	225 (35.0)
White-collar worker in the private sector	248 (38.6)
Other	38 (5.9)

### 2.4. Measures

#### 2.4.1. Well-being

Well-being was assessed using the WHO-5 Well-being index (Topp et al., 2015), which consists of five items, suchs as “I have felt cheerful and in good spirits” with answer options ranging from 1 = *none of the time* to 5 = *all of the time*. Cronbach's α for T1 was 0.87 (T2: α = 0.88, T3: α = 0.90, T4: α = 0.91, T5: α = 0.91, T6: α = 0.92, T7: α = 0.90, T8: α = 0.91).

#### 2.4.2. Time

Time of assessment was coded in weeks (T1–T8), corresponding to the week of participation. The first wave started on 2020–03–24. Accordingly, participants who were recruited in the second week have their initial responses coded as T2.

#### 2.4.3. Predictors of trajectory group membership

##### 2.4.3.1. Telework

Telework was assessed using a single item “Due to the corona crisis I am working from home.” with answer options 1 = *Yes* and 0 = *No/Not anymore*.

##### 2.4.3.2. Work intensification

For work intensification we used three items from the *self-endangering work behaviors* questionnaire by Krause et al. (2015). Sample question: “How often does it usually occur, that you work at a pace, which you felt was straining?” Answer options ranged from 1 = *never* to 5 = *always*. All five items were translated from the German original in tandem with our colleagues from the department of clinical psychology. Cronbach's α for work intensification at T1 was 0.88.

##### 2.4.3.3. Occupational self-efficacy

We used three items from the short occupational self-efficacy scale (Rigotti et al., 2008). Example item: “No matter what comes my way in my job, I'm usually able to handle it.” The answer options ranged from 1 = *strongly disagree* to 5 = *strongly agree*. Cronbach's α for T1 was 0.74.

##### 2.4.3.4. Social support

Social support was measured using a combination of the single item for coworker support from the Copenhagen Psychosocial Questionnaire (COPSOQ) subscales *social support coworkers and superiors*, with the wording “How often do you get help and support from your colleagues?” if participants indicated they had colleagues; together with the item for supervisor's social support, where the wording was “How often do you get help and support from your immediate superior?” (Kristensen et al., 2005). Answer options ranged from 1 = *Never / hardly ever* to 5 = *Always*. Cronbach's α for T1 was 0.75.

##### 2.4.3.5. Decision-making autonomy

We used the subscale for decision-making autonomy from the work design questionnaire (WDQ), consisting of thee items, such as “The job gives me a chance to use my personal initiative or judgement in carrying out the work” with answer options from 1 = *strongly disagree* to 5 = *strongly agree* (Morgeson and Humphrey, 2006). Cronbach's α for T1 was 0.89.

##### 2.4.3.6. Flexitime

Adapted and translated following Büssing and Glaser (2001) and Clark (2002) we used four items to measure working time flexibility. Example items were “I can decide for myself when I work every day” and “I am free to work the hours that are best for my schedule” with answer options ranging from 1 = *strongly disagree* to 5 = *strongly agree*. Cronbach's α for flexitime at T1 was 0.86.

##### 2.4.3.7. Control variables

We controlled for gender, children, cohabitants, age, job tenure, and telework experience; measured at T1. We added gender, children and cohabitants because of the specific situation of the COVID-19 pandemic, in which women were increasingly pushed back into traditional role models and suffered more from loneliness (Zamarro and Prados, 2021; Etheridge and Spantig, 2022). Gender was surveyed with a single item and coded 1 = *female*, 0 = *male*. For *children* and *cohabitants* we offered single items as well: “[...] how many dependent children do you have in your household?” and “How many persons live in your household?”. Furthermore, we included information regarding age and job tenure in the explanatory model, since age and job tenure related differences regarding technostress posed by the COVID-19 pandemic could possibly represent a confounder (Ragu-Nathan et al., 2008). Age and job tenure were measured in years. Telework experience consisted of the single item “Does your employer allow you to do your work from home?” which was coded 1 = *Yes*, 0 = *No*.

### 2.5. Statistical procedure

We used R version 4.1.3 (R Core Team, 2021) and employed a three-step approach as described in Asparouhov and Muthén (2014). We decided to use a GBTM-approach [often referred to as latent class growth analysis (LCGA)] to assess the trajectories of our latent classes. In comparison to the often employed growth mixture modeling (GMM), which assumes the existence of distinct subpopulations, GBTM doesn't estimate within-group variability, and thus intends to approximate trajectories across population members (Nagin and Odgers, 2010). In order to deal with varying numbers of assessment time points as well as differing start times in our survey, we used the R package *LCMM* (version 1.9.3) to specify our models, which uses maximum likelihood estimation (Proust-Lima et al., 2017). To select the best fitting model, we first compared the BIC of several 1-class models with different link-functions (beta, linear, I-splines with varying number of knots) as well as varying specifications of time, allowing estimation of linear, quadratic and cubic trajectories. We used the *gridsearch* function of the package *lcmm* for automatic grid search, with 50 departures from initial values and 100 maximum iterations. In order to assess predictors of group assignment, measured at T1, we applied multinomial regression analysis, using *multinom* from the *nnet* (version 7.3-16) package (Venables and Ripley, 2002).

## 3. Results

### 3.1. Descriptive statistics

Table 2 displays the descriptive statistics and correlations among our study variables at T1. Our outcome well-being, measured with the WHO-5 well-being index, correlates negatively with job demands in terms of work intensification; and positively with personal and job resources. Job demands in terms of work intensification additionally correlate negatively with the job resource flexitime and positively with age and job tenure. As expected personal and job resources appear to be intercorrelated. Interestingly we find a negative relationship between gender and our outcome well-being and the personal resource self-efficacy as well as all job resources, except for social support, indicating slightly lower (in the case of social support higher) values for women in this study. Table A1 in the Appendix displays the correlations of well-being, as well as our focal variables at T1 with the lagged well-being measurements.

**Table 2:** Descriptive statistics and correlations among study variables at T1.

	***M***	***SD***	**01**	**02**	**03**	**04**	**05**	**06**	**07**	**08**	**09**	**10**	**11**	**12**
**Outcome**														
01. Well-being	2.68	1.09												
**Job demands**														
02. Telework (0/1)	0.63	0.48	0.06^***^											
03. Work intensification	2.47	0.78	−0.28^***^	−0.07^***^										
**Personal resources**														
04. Self-efficacy	3.92	0.70	0.37^***^	0.07^***^	−0.06^***^									
**Job resources**														
05. Social support	3.54	0.92	0.15^***^	0.04^***^	−0.05^***^	0.12^***^								
06. Autonomy	3.64	0.92	0.14^***^	0.09^***^	0.03^***^	0.30^***^	0.15^***^							
07. Flexitime	3.75	1.02	0.19^***^	0.44^***^	−0.13^***^	0.15^***^	0.15^***^	0.34^***^						
**Control variables**														
08. Age	39.63	12.81	0.06^***^	−0.05^***^	0.21^***^	0.18^***^	0.02^***^	0.11^**^^*^	0.01^***^					
09. Gender	0.71	0.46	−0.14^***^	−0.11^**^^*^	0.05^***^	−0.16^***^	0.08^*^^**^	−0.11^**^^*^	−0.09^*^^**^	−0.07^***^				
10. Job tenure	8.12	9.56	0.01^***^	−0.09^*^^**^	0.19^***^	0.05^***^	0.00^***^	0.02^***^	−0.08^*^^**^	0.65^***^	−0.08^*^			
11. Children	1.77	0.89	−0.05^***^	−0.11^***^	0.03^***^	0.03^***^	−0.07^***^	0.00^***^	−0.10^***^	0.18^*^^*^	−0.10^*^	−0.02^*^		
12. Cohabitants	2.48	1.46	0.02^***^	−0.04^***^	−0.04^***^	0.01^***^	−0.05^***^	−0.05^***^	−0.05^***^	0.08^***^	−0.07^*^	0.08^*^	0.48^***^	
13. Telework experience	0.59	0.49	0.08^*^^**^	0.60^***^	−0.04^***^	0.03^***^	0.04^***^	0.13^**^^*^	0.40^***^	0.01^***^	−0.08^*^	−0.09^*^	−0.03^***^	0.00

*M* = mean; *SD* = standard deviation;

*** *p* < 0.001;

** *p* < 0.01;

* *p* < 0.05; *N* = 642. Telework: 1 = teleworking; Gender: 1 = female; Age and tenure in years. Telework experience: 1 = yes.

### 3.2. Trajectories of well-being (H1)

**Figure 2:**
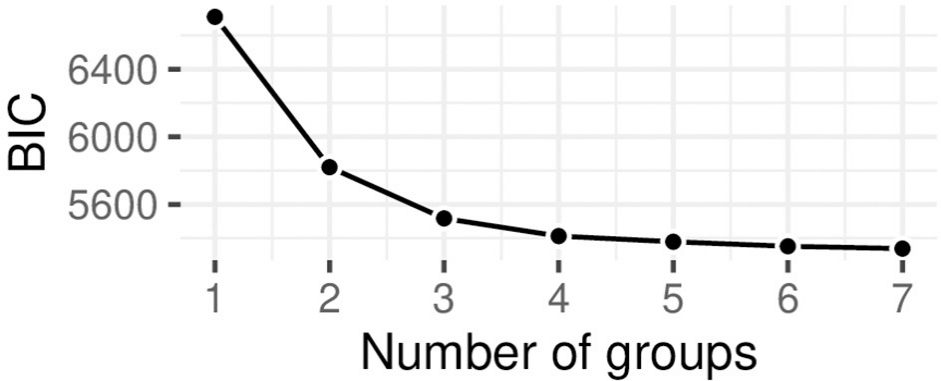
Elbow plot of BIC values for model selection.

Table 3 displays the fit indices and entropy of our estimated models. Three groups should fit the data well enough, as can be seen in the elbow plot in Figure 2. Entropy was slightly lower from the two-class solution (0.76) but still acceptable with 0.75. The small decrease in BIC doesn't justify the risk of overfitting, by choosing a model comprised of more classes. Additionally we looked at the posterior probability of class membership assignment, which was above 0.8 on average for each of the classes.

**Table 3:** Model indices of 1–7 classes for well-being.

**G**	**npm**	**AIC**	**BIC**	**SABIC**	**Entropy**	**%Class1**	**%Class2**	**%Class3**	**%Class4**	**%Class5**	**%Class6**	**%Class7**
1	6	6,682.52	6,709.31	6,690.26	1.00	100.00						
2	9	5,780.00	5,820.18	5,791.61	0.76	52.65	47.35					
3	12	5,463.59	5,517.17	5,479.07	0.75	22.59	32.40	45.02				
4	15	5,345.86	5,412.83	5,365.21	0.72	37.23	35.20	15.89	11.68			
5	18	5,298.52	5,378.89	5,321.74	0.73	35.83	2.80	18.54	11.06	31.78		
6	21	5,258.58	5,352.34	5,285.66	0.74	34.42	29.60	13.24	1.40	2.80	18.54	
7	24	5,231.42	5,338.57	5,262.37	0.74	18.69	33.96	27.88	2.80	1.71	13.55	1.4

G = number of groups; npm = number of iterations; AIC = Aikaike Information; BIC = Bayesian Information Criteria; SABIC = Sample Size Adjusted Bayesian Information Criteria.

**Figure 3:**
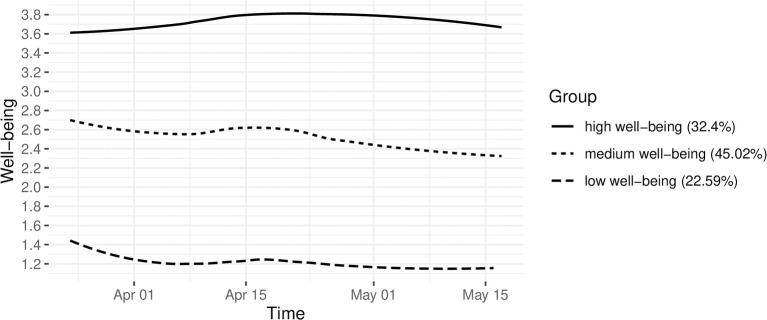
Group trajectories of observed average well-being scores.

The three-class model clearly differentiated three groups, which are displayed in Figure 3 using observed values with smoothed average scores. *Group: low level of well-being* consisted of 22% of the sample and is represented by an initially ever so slightly decreasing level of well-being as measured by the WHO-5 well-being index. Participants in this group on average had a higher level of well-being at the earlier time points, which deteriorated in the first couple of weeks, to slightly improve in the middle of April and consequently remain low throughout the the rest of the study. *Group: medium level of well-being* had the highest percentage of participants with 45% and is comprised of individuals with on average consistently medium levels of well-being. Similar to the low level of well-being group, we find an initial slight decline of well-being in this group, which recovers a little bit in the middle of April, to then deteriorate across the rest of the measurement time points. *Group: high level of well-being* consistently showed the highest level of well-being on average. This group displayed a continuous improvement of well-being up until May. Thereafter it declined slightly, but still stayed above the initial levels until the end of our study.

### 3.3. Group membership predictors, main effects (H2–H3, H5s–Hb)

Table 4 displays the result of our multinomial regression analysis, predicting trajectory group membership (AIC = 1, 252.32).

**Table 4:** Multinomial logistic regression of group membership at T1, reference group: high well-being.

	**Reference group = high well-being**	
	**Medium well-being**	**Low well-being**
Intercept	3.06 (1.41)^*^	5.47 (1.76)^**^
Telework (yes = 1)	1.61 (1.84)	0.38 (2.25)
Work intensification	1.08 (0.25)^***^	1.70 (0.32)^***^
**Personal resources**		
Occupational self-efficacy	−0.93 (0.29)^**^	−2.02 (0.37)^***^
**Job resources**		
Social support	−0.05 (0.19)	−0.22 (0.24)
Decision-making autonomy	0.04 (0.20)	0.32 (0.26)
Flexitime	−0.24 (0.19)	−0.34 (0.23)
**Interactions**		
Telework × Occupational self-efficacy	0.50 (0.36)	1.19 (0.44)^**^
Telework × Social support	−0.10 (0.23)	−0.28 (0.29)
Telework × Decision-making autonomy	−0.24 (0.25)	−0.49 (0.32)
Telework × Flexitime	−0.16 (0.25)	−0.04 (0.31)
Telework × Work intensification	−0.63 (0.30)^*^	−0.68 (0.38)
**Control variables**		
Age	−0.02 (0.01)	−0.02 (0.01)
Gender (female = 1)	0.19 (0.22)	0.13 (0.29)
Job tenure (years)	0.00 (0.01)	−0.03 (0.02)
Children	0.01 (0.14)	0.61 (0.21)^**^
Cohabitants	−0.06 (0.08)	−0.39 (0.15)^**^
Telework experience (yes = 1)	−0.06 (0.25)	−0.22 (0.33)

*** *p* < 0.001;

** *p* < 0.01;

* *p* < 0.05.

#### 3.3.1. Hypothesis H2

We expected telework to be associated with trajectory profiles which indicate lower levels of well-being. No significant associations could be identified, neither for the change from high well-being group membership to low well-being group membership (β = 0.38, *p* = 0.867), nor for change from high well-being group membership to medium well-being group membership (β = 1.61, *p* = 0.380).

#### 3.3.2. Hypothesis H3

We assumed that the job demand work intensification would be associated with trajectory profiles which indicate lower levels of well-being. We found that work intensification was significantly related to well-being: with high well-being as reference, an increase in work intensification would lead to an increase of the likelihood of being a member of the low well-being group (β = 1.70, *p* < 0.001); this similarly applied to the likelihood of being a member of the medium well-being group (β = 1.08, *p* < 0.001).

#### 3.3.3. Hypothesis H5a

We assumed that personal resources would be associated with trajectory profiles which indicate higher levels of well-being. We found that the personal resource occupational self-efficacy was significantly related to well-being: with high well-being as reference, an increase in occupational self-efficacy would lead to a decrease of the likelihood of being a member of the low well-being group (β = −2.02, *p* < 0.001); this similarly applied to the likelihood of being a member of the medium well-being group (β = −0.93, *p* = 0.002).

#### 3.3.4. Hypothesis H5b

We hypothesized that job resources would be related to well-being group membership. No significant associations could be identified for any of the job resources included in this study, neither for the likelihood of belonging to the low well-being group (social support: β = −0.22, *p* = 0.360; decision-making autonomy: β = 0.32, *p* = 0.208; flexitime: β = −0.34, *p* = 0.139), nor for the likelihood of belonging to the medium well-being group (social support: β = −0.05, *p* = 0.794; decision-making autonomy: β = 0.04, *p* = 0.863; flexitime: β = −0.24, *p* = 0.205).

### 3.4. Group membership predictors, moderator effects (H4, H6a–H6b)

#### 3.4.1. Hypothesis H4

We expected the influence of telework on trajectory profile membership to be moderated by the level of the job demand work intensification. For the low well-being group the interaction between telework and work intensification wasn't significant (telework × work intensification: β = −0.68, *p* = 0.070). A significant association was found for the medium well-being group regarding the interaction between telework and work intensification with a coefficient of β = −0.63, *p* = 0.032.

#### 3.4.2. Hypothesis H6a

Only the interaction term of the personal resource occupational self-efficacy with telework had a significant association with the likelihood of belonging to the low well-being group (β = 1.19, *p* = 0.007). For the medium well-being group the interaction between telework and personal resources or job resources was not significant (telework × occupational self-efficacy: β = 0.50, *p* = 0.163).

#### 3.4.3. Hypothesis H6b

Interactions of telework with all of the examined job resources were non-significant for the low well-being group (telework × social support: β = −0.28, *p* = 0.335; telework × decision-making autonomy: β = −0.49, *p* = 0.129; telework × flexitime: β = −0.04, *p* = 0.902); as well as the medium well-being group (telework × social support: β = −0.10, *p* = 0.662; telework × decision-making autonomy: β = −0.24, *p* = 0.349; telework × flexitime: β = −0.16, *p* = 0.524).

#### 3.4.4. Control variables

For our control variables *age, gender, tenure*, and *telework experience* we found no significant relationship to group membership. The likelihood for members of the high well-being group of belonging to the low well-being group increased as the number of *children* increased (β = 0.61, *p* = 0.003) and decreased the more *cohabitants* one had (β = −0.39, *p* = 0.010).

#### 3.4.5. Graphical interpretation

**Figure 4:**
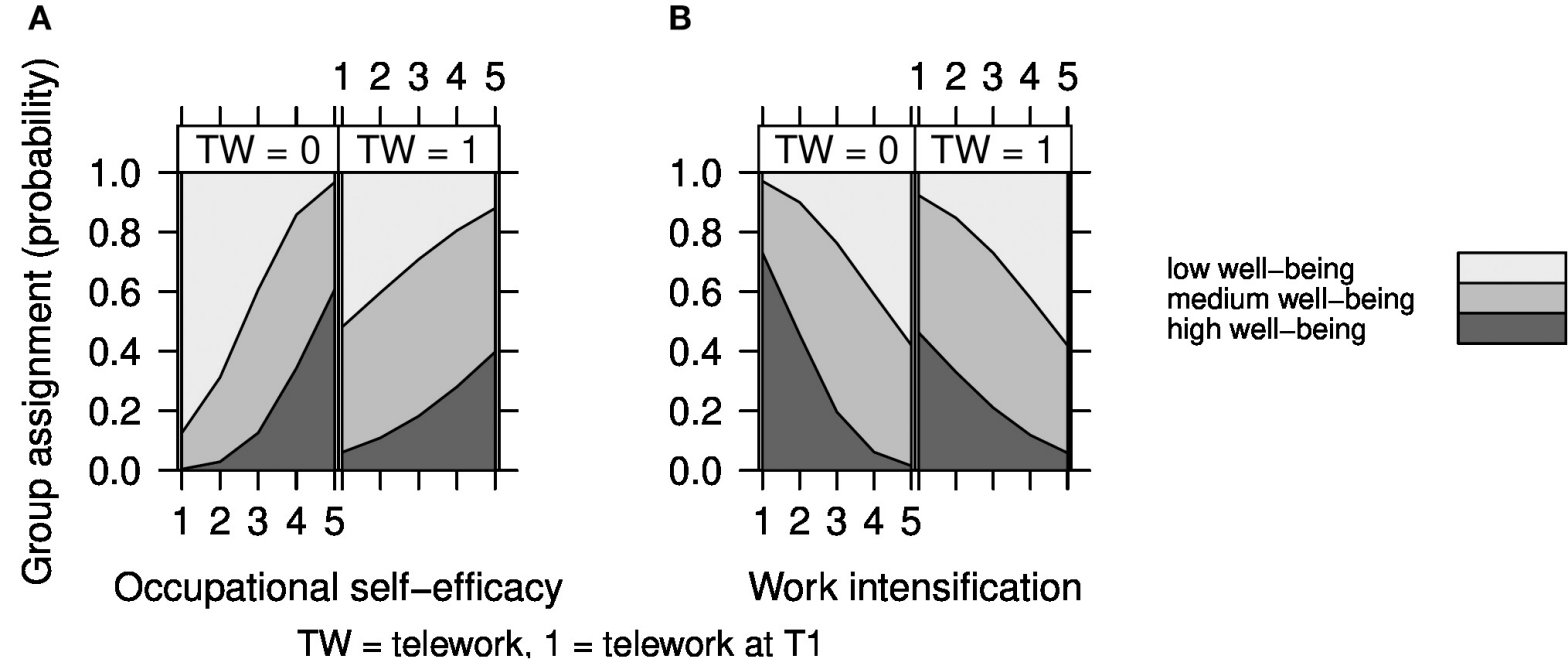
The interaction effect of **(A)** telework × occupational self-efficacy and **(B)** telework × work intensification on well-being.

To pinpoint the location and direction of our interaction effects, we plotted the group membership probability in function of our interaction terms in Figure 4. For the interaction of telework × occupational self-efficacy on well-being it becomes clear that, as occupational self-efficacy increases for participants without access to telework at T1 the probability of being a member of the low well-being group decreases. Having access to telework similarly decreases the probability of being part of the low well-being group even with low availability of occupational self-efficacy.

For the interaction of telework × work intensification on well-being we find that without availability of telework at T1 the likelihood of assignment to the high well-being group becomes rather slim as work intensification increases. Given the availability of telework the impact of increased work intensification isn't as pronounced: the likelihood of being a member of the high well-being group doesn't fall as sharply as without telework.

## 4. Discussion

This study intended to investigate how the onset of telework affected trajectories of mental well-being of working people and which resources were particularly helpful for coping with the new telework demands. We were able to identify three well-being groups (high, medium and low well-being), each of which showed a fairly static course over the survey period. In particular, the high well-being group tended to show a slight improvement in well-being, while the two groups of medium and low well-being deteriorated slightly.

Regarding the predictors of well-being trajectory group membership, which we measured at the initial time points, only the job demand work intensification had a significant relationship with trajectory group membership, pointing toward higher levels of work intensification being associated with lower levels of mental well-being. For the onset of telework we couldn't confirm a direct effect on well-being group membership. The personal resource occupational self-efficacy showed a direct, significant effect indicating a positive relationship between high well-being and occupational self-efficacy. No significant association were found for either of the examined job resources (i.e., social support, job autonomy and flexitime).

For our moderator hypotheses, we found an opposite than assumed buffering effect of telework. Having access to telework increased the likelihood of being a member of the medium or high well-being group even when occupational self-efficacy is low. That is, the availability of telework could compensate for a lack of occupational self-efficacy in regard to well-being group membership. Similar results were found for the interaction of telework with work intensification: the effects of work intensification were buffered by the availability of telework. Having access to telework increased the likelihood of being a member of the high well-being group even when work intensification is high. Again no significant interaction effects were found for telework with either of the examined job resources.

### 4.1. Theoretical implications

Building upon the *accumulation* and *adjustment* models of stress (see Zapf et al., 1996) we had assumed to identify varying trajectory profiles of mental well-being, but had to discard all of them, except for a rather stagnant, albeit slightly improving trajectory of well-being for the high well-being group and deteriorating trajectories for the medium and low well-being groups. Using the *accumulation* and *adjustment* models to explain these findings would suggest, that during the initial weeks of the pandemic, the participants of this study did not experience much of an increase in stressors or had sufficient resources to adjust to the new situation (Zapf et al., 1996). We had expected to find more variation regarding trajectories of mental well-being, given that high day-to-day fluctuations of mental well-being have been reported (Totterdell et al., 2006). It is interesting to see that especially the persons with limited well-being suffered further losses, while the group with high well-being gained slightly in terms of well-being. This may be explained by the conservation of resources (COR) theory (see Hobfoll, 1989), which suggests, that individuals with more availability of resources are more likely to receive additional resources, while those with fewer resources are more likely to be threatened by resource-loss, similar to the Matthew effect: “For to every one who has will more be given, and he will have abundance; but from him who has not, even what he has will be taken away.” (Matthew 25:29).

Our data did not support our hypothesis that the onset of telework is a job demand, which we had assumed to due its sudden implementation and reports of increased job demands due to teleworking during the COVID-19 pandemic (Wang et al., 2021). On the contrary, it turned out that in the initial phase of the COVID-19 pandemic, telework was a resource that could buffer the consequences of job demands in terms of work intensification and, in part, compensate for a lack of personal resources. Possibly, previous literature on telework was positively biased in the sense that mainly workers were included, who desired to telework and had favorable conditions (Delanoeije and Verbruggen, 2020); and it is not entirely clear whether telework could not also serve as a demand instead of a resource. Our article contributes to this question, because due to the forced transition to telework, we were able to survey people whose job and personal conditions were not necessarily ideal for it. This perception of telework as a job resource in the early stages of the COVID-19 pandemic is also reflected by the trend of many employees calling for a continuation of telework arrangements beyond the pandemic, which holds especially true for younger workers, as well as those with higher information and communication technology (ICT) use and those with positive experiences with telework (Georgescu et al., 2021). Additionally a recent survey found that 54% of 16,000 surveyed workers, across industries and countries, would consider quitting their job if certain amenities they experienced during the pandemic, like working from home and work time flexibility, were to be retracted (Ernst & Young, 2021; Melin and Egkolfopoulou, 2021). This classification of telework as a job resource is also supported by the significant interaction of telework with the personal resource occupational self-efficacy on well-being: the compensation of no telework availability through occupational self-efficacy is in line with and supports the assumptions of the job demands-resources (JD-R) model, according to which personal resources can compensate for missing job resources (Bakker and Demerouti, 2017).

The absence of significant main effects for the remaining job resources and their interactions with telework may possibly be explained by the specific circumstances brought upon by the COVID-19 pandemic: the effects of perceived autonomy, social support, or flexitime at the first survey time point may pale behind the importance of self-regulatory strategies in terms of occupational self-efficacy, as this may have been essential for transitioning and adapting to the pandemic situation. Similar results for teleworkers were found in France, where the job resources autonomy and organizational support didn't have as much of an impact on adjustment to telework as expected (Carillo et al., 2021). This would suggest that employees' belief in their ability to perform well in their job, regardless of the work environment, is more influential on their well-being than the resources provided by their job. The relevance of personal resources for well-being during the pandemic is in accordance with the literature (e.g., Cotel et al., 2021; Joie-La Marle et al., 2021). The lack of a significant effect of job resources on mental well-being during pandemic in this study may also inform the JD-R model, by highlighting the importance of considering personal resources such as self-efficacy in addition to job resources (Xanthopoulou et al., 2007). It is important to caution against overinterpreting the findings of our study as it was conducted during a specific situation the pandemic.

Nevertheless we found a significant association of some of our control variables with well-being group membership, in the direction and context expected from the literature (i.e., children increasing the likelihood of being in the low well-being group; cohabitants decreasing it; Zamarro and Prados, 2021; Etheridge and Spantig, 2022), which speaks for the validity of our results, which held under the addition of these variables.

Although we had to discard telework as a job demand and rather found it being a job resource, we are able to add to the literature about personal resources in the JD-R model whose moderating relationship between job resources and the health improvement path had been recognized by Xanthopoulou et al. (2007), but not tested. Similar to our results, personal resources (i.e., optimism) were found to buffer the negative effect of low job resources on work engagement (Salminen et al., 2014).

What did we learn? Contrary to our assumptions, our study shows that telework is a job resource even in in its unfamiliar initial phase. Moreover we add to the JD-R literature by demonstrating positive effects of job and personal resources on trajectories of well-being over time.

### 4.2. Limitations and future research

Of course, our study has limitations that we would like to point out. We must caution about generalizing our results to non-pandemic periods and, although we surveyed longitudinally, we cannot specify cause-effect relationships because we examined only the effect of variables at T1 on well-being trajectory group membership. A clear strength of the study is that it captures well the onset of pandemic-induced changes, given that we started our survey immediately when lockdown mandates came to effect in Germany. It is conceivable that parts of our findings are due to influences of the particular situation rather than finding their genesis in the general onset of telework. Since the data analyzed here was limited to Germany, our research's applicability to other countries can't be assumed. This is equally true for the german work force in general. We can't rule out bias due to our retrospective approach of querying well-being (i.e., “How did you feel in the past week?”), as well as common method bias and using self-ratings instead of objective measures of well-being (Podsakoff et al., 2003; Schmier and Halpern, 2004). Additionally it is important to note that the majority of the convenience sample studied here was well educated, thus we can't assume absence of a socioeconomic bias either.

Future research should try to replicate our study design under non-pandemic conditions. Furthermore, it would be conceivable to use an experimental design in which long-term well-being is examined by means of occupational self-efficacy training, with and without the offer of telework, in order to be able to better analyze the individual effect facets.

### 4.3. Practical implications

Work intensification has been shown to be a stressor that is predictive of psychological well-being. Our results suggest that telework may contribute to improved coping with intensified work conditions. Telework can buffer the effects of work intensification and has a positive impact on well-being trajectories. In practice, it could be useful to grant stressed employees additional (or in principle) teleworking time to cope with special workloads and demands.

In addition, the study also shows that training of occupational self-efficacy can be useful to deal with special situations (e.g., when telework is not possible), since in our study the personal resource occupational self-efficacy in contrast to job resources had a sustainable effect on well-being trajectory group membership. A promising approach here could be, for example, interventions aimed at increasing psychological capital, a construct which, in addition to self-efficacy, also includes hope, optimism, and resilience (Luthans et al., 2010).

### 4.4. Conclusion

In conclusion, our study has shown that telework can have a positive impact on well-being by buffering the negative effects of work intensification. The results indicate that access to telework, where possible, can lead to improved outcomes in terms of well-being and can help employees cope with special situations such as the COVID-19 pandemic.

Furthermore, our study revealed that occupational self-efficacy is a personal resource that has a direct measurable effect beyond traditional job resources in the early period of the pandemic. The results suggest that training in occupational self-efficacy can be beneficial in helping employees deal with the unique stressors and challenges brought about by the pandemic. This highlights the importance of considering both job and personal resources in understanding employee well-being during the pandemic.

It is important to keep in mind, however, that the results of this study were obtained during a specific situation, the COVID-19 pandemic, and should not be generalized to other teleworking situations.

## Data availability statement

The raw data supporting the conclusions of this article will be made available by the authors, without undue reservation.

## Ethics statement

The studies involving human participants were reviewed and approved by Institute of Psychology of the University of Duisburg-Essen's Ethics Committee. The patients/participants provided their written informed consent to participate in this study.

## Author contributions

FK and AM contributed to conception and design of the study. FK performed data collection, statistical analysis, and drafted the manuscript. AM provided the critical revision of the manuscript. Both authors have read and approved the submitted version.

## Appendix

**Table A1:** Correlations among study variables and well-being at different time points.

	**Well-being**				
	**T1**	**T2**	**T3**	**T4**	**T5**
**Well-being**					
T1	1.00^***^				
T2	0.70^***^	1.00^***^			
T3	0.67^***^	0.78^***^	1.00^***^		
T4	0.61^***^	0.69^***^	0.78^***^	1.00^***^	
T5	0.62^***^	0.73^***^	0.76^***^	0.80^***^	1.00^***^
**Predictors at T1**					
Telework (0/1)	0.06	0.01	0.07	0.06	0.03
Work intensification	−0.28^***^	−0.23^***^	−0.23^***^	−0.31^***^	−0.35^***^
Self−efficacy	0.37^***^	0.32^***^	0.25^***^	0.22^***^	0.30^***^
Social support	0.15^***^	0.17^***^	0.14^**^	0.17^**^	0.15^**^
Autonomy	0.14^***^	0.11^*^	0.10^*^	0.00	0.06
Flexitime	0.19^***^	0.12^**^	0.18^***^	0.15^**^	0.15^*^
Age	0.06	0.04	0.02	−0.06	−0.07
Gender	−0.14^***^	−0.06	−0.05	−0.05	−0.09
Job tenure	0.01	0.00	0.02	−0.06	−0.08
Children	−0.05	−0.03	0.03	0.04	0.12
Cohabitants	0.02	−0.02	−0.03	0.02	0.00
Telework experience	0.08	0.02	0.06	0.08	0.02

*** *p* < 0.001;

** *p* < 0.01;

* *p* < 0.05; Telework: 1 = teleworking; Gender: 1 = female; Age and tenure in years. Telework experience: 1 = yes.
